# Neural stem/progenitor cell therapy for Alzheimer disease in preclinical rodent models: a systematic review and meta-analysis

**DOI:** 10.1186/s13287-022-03231-1

**Published:** 2023-01-05

**Authors:** Zijing Zhou, Ben Shi, Yaxing Xu, Jinyu Zhang, Xin liu, Xinghong Zhou, Baofeng Feng, Jun Ma, Huixian Cui

**Affiliations:** 1 Hebei Medical University-National University of Ireland Galway Stem Cell Research Center, Hebei Medical University, Shijiazhuang, 050017 Hebei Province China; 2 Hebei Research Center for Stem Cell Medical Translational Engineering, Hebei Medical University, Shijiazhuang, 050017 Hebei Province China; 3 Human Anatomy Department, Hebei Medical University, Shijiazhuang, 050017 Hebei Province China

**Keywords:** Alzheimer disease, Neural stem/progenitor cell, Rodent models, Systematic review and meta-analysis

## Abstract

**Background:**

Alzheimer’s disease (AD) is a common progressive neurodegenerative disease characterized by memory impairments, and there is no effective therapy. Neural stem/progenitor cell (NSPC) has emerged as potential novel therapy for AD, and we aim to explore whether neural stem/progenitor cell therapy was effective for rodent models of AD.

**Methods:**

We searched PubMed, Embase, Cochrane Library and Web of Science up to December 6, 2022. The outcomes included cognitive function, pathological features and BDNF. The GetData Graph Digitizer software (version 2.26) was applied to extract numerical values, and RevMan 5.3 and Stata 16 were used to analyze data. The SYRCLE risk of bias tool was used to assess study quality.

**Results:**

We evaluated 22 mice studies and 8 rat studies. Compared to control groups, cognitive function of NSPC groups of both mice studies (SMD =  − 1.96, 95% CI  − 2.47 to  − 1.45, *I*^2^ = 75%, *P* < 0.00001) and rat studies (SMD =  − 1.35, 95% CI − 2.11 to  − 0.59, *I*^2^ = 77%, *P* = 0.0005) was apparently improved. In mice studies, NSPC group has lower A*β* deposition (SMD =  − 0.96, 95% CI  − 1.40 to  − 0.52, *P* < 0.0001) and *p*-tau level (SMD =  − 4.94, 95% CI  − 7.29 to  − 2.95, *P* < 0.0001), higher synaptic density (SMD = 2.02, 95% CI 0.50–3.55, *P* = 0.009) and BDNF (SMD = 1.69, 95% CI 0.61–2.77, *P* = 0.002). Combined with nanoformulation (SMD =  − 1.29, 95% CI  − 2.26 to  − 0.32, *I*^2^ = 65%, *P* = 0.009) and genetically modified (SMD =  − 1.29, 95% CI  − 1.92 to  − 0.66, *I*^2^ = 60%, *P* < 0.0001) could improve the effect of NSPC. In addition, both xenogeneic and allogeneic transplant of NSPC could reverse the cognitive impairment of AD animal models.

**Conclusions:**

Our results suggested that NSPC therapy could improve the cognitive function and slow down the progression of AD. Due to the limitations of models, more animal trials and clinical trials are needed.

**Supplementary Information:**

The online version contains supplementary material available at 10.1186/s13287-022-03231-1.

## Introduction

Alzheimer disease (AD) is a common, progressive, and devastating neurodegenerative disease. The pathological features of the disease are the presence of extracellular amyloid-*β* (A*β*)-containing senile plaques and intracellular hyperphosphorylated tau-containing neurofibrillary tangles (NFT), neuroinflammation, synaptic loss and neuronal death, neocortical atrophy and the progressive deterioration of cognitive function [1, 2]. AD can be divided into familial Alzheimer disease (FAD) and sporadic AD (SAD) among the genetic factors. Most patients with Alzheimer’s disease (> 95%) have the sporadic form, which is characterized by a late onset (80–90 years of age) and is the consequence of the failure to clear the amyloid-*β* (A*β*) peptide from the interstices of the brain [3]. Familial Alzheimer’s disease (FAD) presents basic similarities to sporadic AD, but with important differences. Onset is in mid-life or earlier, and the genetics follows a dominant Mendelian pattern, with 100% penetrance in most pedigrees [4]. The pathogenesis of AD is complex, involving multiple molecular signaling pathways. Cholinergic deficiency, amyloid beta (A*β*) toxicity, tau protein hyperphosphorylation, synaptic dysfunction, oxidative stress, and neuroinflammation, were proposed to be responsible for AD development [5]. In 2018, Alzheimer’s Disease International estimated a dementia prevalence of about 50 million people worldwide, projected to triple in 2050, with two-thirds living in low-income and middle-income countries.

Today, only five drugs have been approved by the FDA for AD treatment: donepezil, rivastigmine, galantamine, tacrine and memantine. The first four drugs are acetylcholinesterase inhibitors (AChEIs), while the last one is an *N*-methyl-d-aspartate receptor (NMDAR) antagonist [6]. Clinical studies show some other approaches to AD, such as acupuncture, behavioral training and brain stimulation, including deep brain stimulation (DBS) [7], repetitive transcranial magnetic stimulation (rTMS) [8] and transcranial electrical stimulation (tDCS and tACS) [8, 9]. But current treatments are unable to achieve satisfactory therapeutic outcomes, new treatments are urgently needed.

In recent years, stem cell therapy has received growing attention as a potential regenerative therapy for neurodegenerative diseases including AD due to regeneration of neural tissue, stabilizing the neuronal networks, providing neurotrophic support and alleviating neurodegeneration at different neuronal circuitry levels [10]. In clinical trials, researchers are conducting the safety and efficacy of Mesenchymal Stem Cells and Autologous Adipose Tissue Derived Mesenchymal Stem Cells. Phase I clinical trials of human umbilical cord blood derived mesenchymal stem cells and Longeveron Mesenchymal Stem Cells preliminary prove that MSC therapy was feasible, relatively and sufficiently safe and well tolerated [11, 12]. As for other animal models, there are more types of stem cells—induced pluripotent stem cells (iPSCs), neural stem cells (NSCs), mesenchymal stem cells (MSCs) and embryonic stem cells (ESCs). Neural stem/progenitor cells (NSPCs) are the multipotent stem cells that are capable of proliferation, self-renewal and generation of new neurons, astrocytes and oligodendrocytes [13]. NSPCs were used for some animal models, which have evaluated the safety and effectiveness of NSPC therapy. But there is no meta-analysis to evaluate the efficacy and synthesize evidence of NSPC therapy in AD models. Therefore, the aim of this systematic review and meta-analysis is to assess the efficacy of NSPC therapy of experimental AD rodents, and our study will provide support for clinical treatment of NSPC for AD.

## Methods

### Data sources and search strategy

Four database (PubMed, Embase, Web of Science and Cochrane Library) were searched for experimentally controlled studies of the effect of NSPC therapies on AD models from their inception to December 6, 2022. The search strategy used a combination of terms from medical subject headings (MeSH) and free-text keywords. The subject headings were "Alzheimer Disease" AND "Neural Stem Cells" AND "Mice" OR "Rats." Combined with free words: (Alzheimer Dementia OR Dementia, Alzheimer OR Alzheimer’s Disease OR Alzheimer Syndrome) AND (Neural Stem Cell OR Neural Progenitor Cell OR neural stem/progenitor cell) AND (mouse OR rat). Manual search and other methods were used to identify other relevant articles. Information of detailed search strategy is shown in Additional file 1: Table S1.

Criteria for consideration and extraction

- *Inclusion criteria* (1) AD mice/rats treated by NSPCs; (2) Studies provided data about MWM or A*β* level; (3) Studies were published in English.
- *Exclusion criteria* (1) No in vivo texting; (2) Review or conference abstract; (3) No NSPC group or control group; (4) No outcome or incomplete data.

### Study selection

The literature retrieved from each database was imported into the NoteExpress, and the duplicated papers were removed. Then, titles and abstracts were scrutinized to determine the eligible studies after excluding the irrelevant articles. Then, full-text papers were obtained reviewed for the final eligibility according to the inclusion and exclusion criteria stated above. Two researchers independently select the studies, and a third researcher was consulted to resolve any disagreements.

### Data extraction and quality assessment

Two researchers independently evaluated article quality and extracted data, and disagreements were addressed by discussion with a third reviewer. We extracted the following data from each study: first author, year, location, sex, species, weight and year of animals, method of AD model induction, source of NSPC, dose of cells, way and location of administrated, groups of trials, assessment time, immunosuppression or not and outcome. If data were only shown by graphs, the GetData Graph Digitizer software (version 2.26) was applied to extract numerical values. When SD was not reported, it was calculated by √N × SE, and N means the sample size. If the required information was not obtained, the study was deleted. The SYRCLE risk of bias tool was used to evaluate the quality of animal studies [14].

### Statistical analysis

Cochrane Collaboration Software RevMan 5.3 and Stata 16 were used to analyze data. The combined effect size was calculated as standardized mean difference (SMD) with 95% confidence interval (CI) between treatment group and control group. Heterogeneity was statistically evaluated by *I*^2^value, *I*^2^ ≤ 50% indicated homogeneity and fixed-effect models were employed, or random-effect models were used instead. Subgroup analyses were performed to indicate statistical significance. Publication bias was investigated by visual inspection of funnel plots. All tests were two-sided, and *P* < 0.05 was considered to indicate statistical significance.

## Results

### Search results

**Fig. 1:**
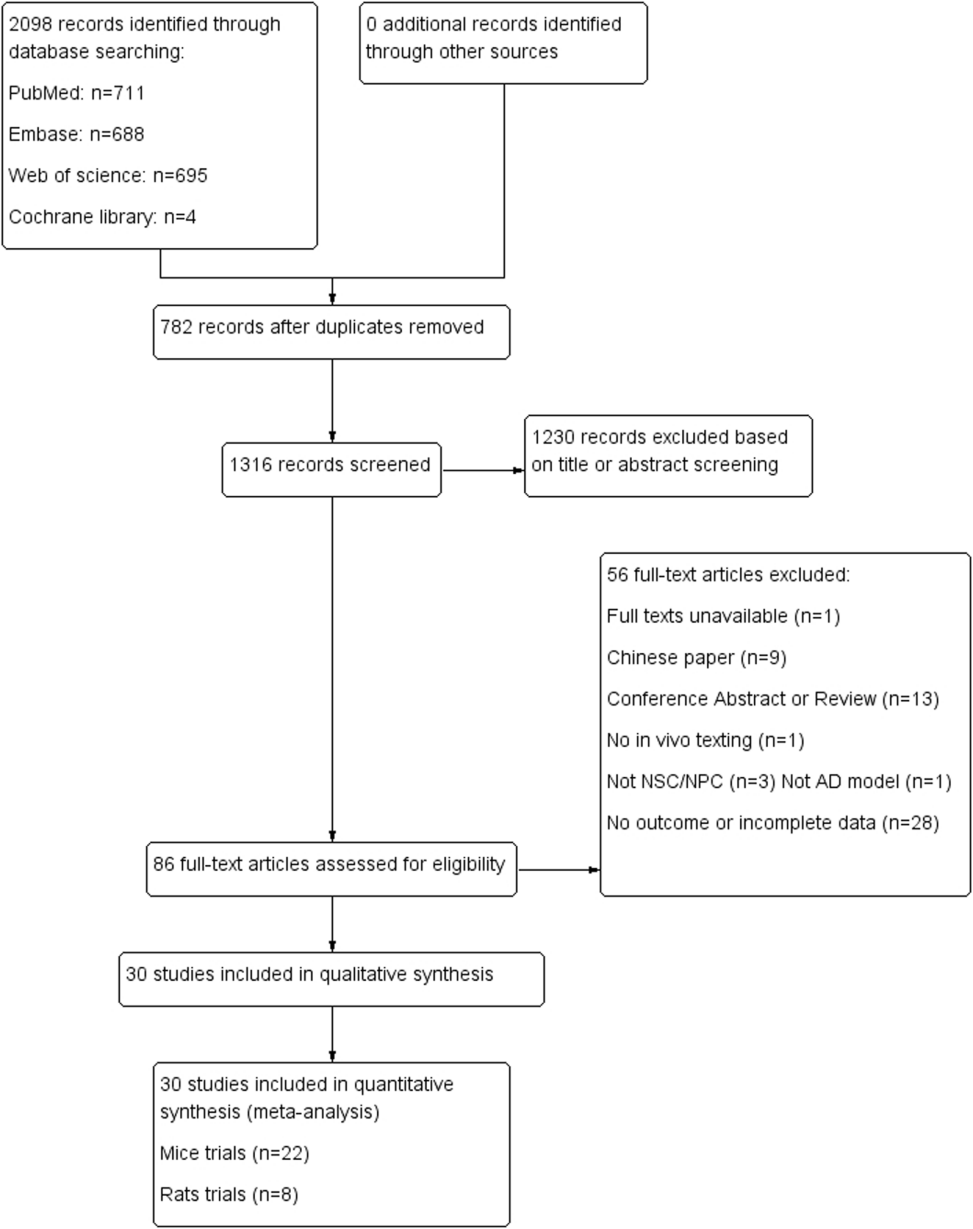
PRISM flowchart of study selection process. A total of 2098 records were retrieved; after application of the inclusion criteria, 22 mice studies and 8 rat studies were included

A total of 2098 articles were initially retrieved from 4 databases, and 1316 records were obtained after removing 782 duplicates. Then after screening titles and abstracts, 86 full-text articles were assessed for eligibility. Fifty-six of them were excluded because of full text unavailable, Chinese paper, conference abstract or review, no in vivo texting, no NSPC or AD model and no outcome or incomplete data. Finally, 22 mice trials [15–36] and 8 rat trials [37–44] were selected (Fig. 1). Funnel plots were used to evaluate publication bias (Additional file 2: Fig. S1).

### Study characteristics and quality

**Fig. 2:**
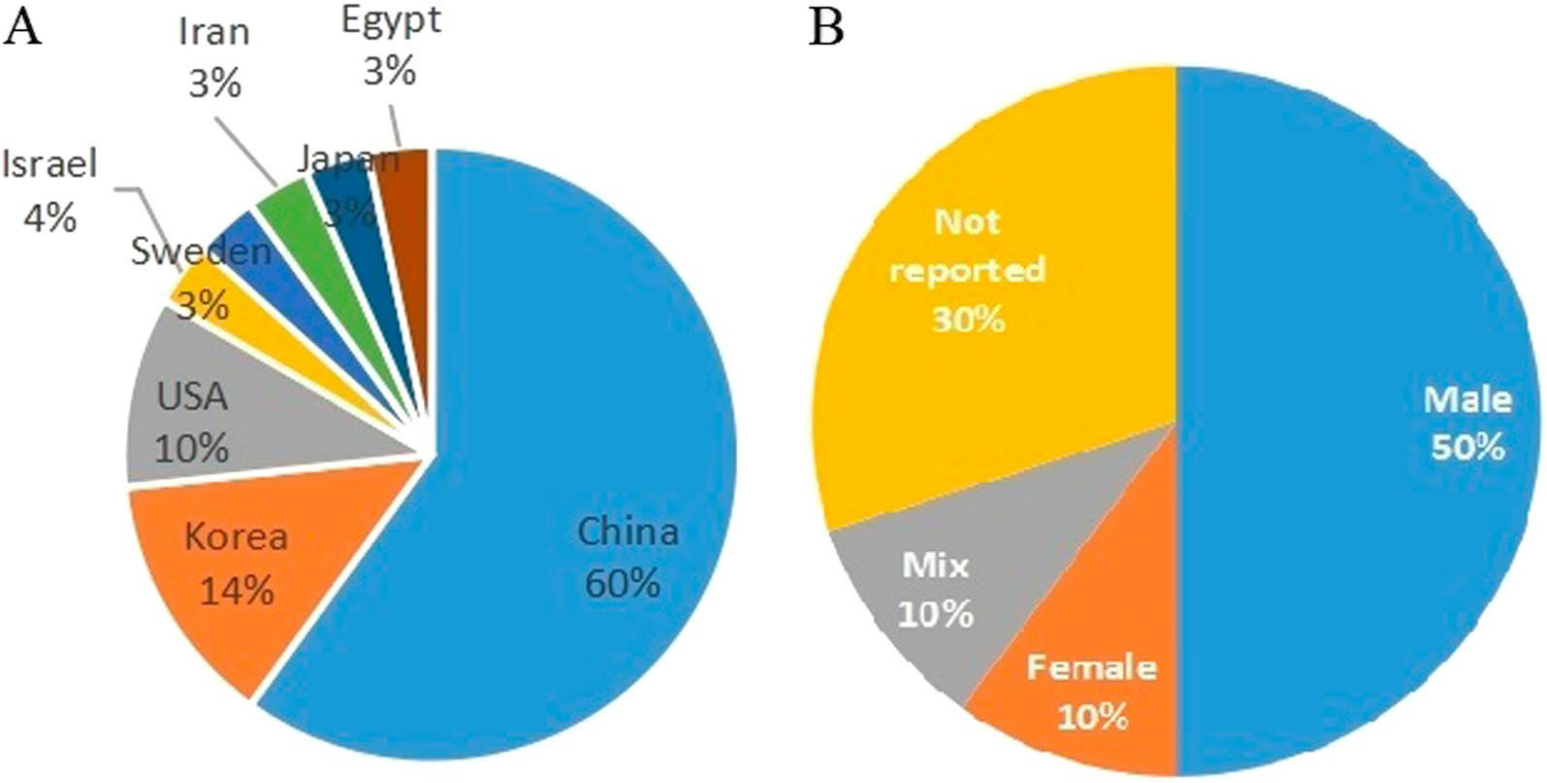
Mapping of study characteristics included in the systematic review. **A** Location of studies. **B** Gender of animals

Of 30 studies, 22 were mice models and 8 were rat models. The location of studies included China, Korea, the USA, Israel, Sweden, Iran, Japan and Egypt (Fig. 2a). The gender of the experimental animal of all studies included only male, or only female, or the mixed, except for 9 studies with no statements (Fig. 2b). Of all mice models, APPswe/PS1dE9 mice were used in 11 studies, Tg2576 mice and APP/PS1/tau 3 × Tg AD mice were used in 3 studies, SAMP8 mice were used in 2 studies, and Tg-tau mice, NSE/APPsw transgenic mice and ICR mice infused with ibotenic acid were used in other studies. Sixteen studies used mice NSPC, 6 studies used human NSPC, and 3 studies used immunosuppression. As for rat model, 6 studies used SD rats and 2 used Wistar rats, while the method of AD is different, such as infusing AF64A solution, A*β*, okadaic acid (OA), IgG-saporin, ibotenic (IBO) acid and nucleus basalis of Meynert (nbM) lesioning. Four studies used rat NSPC, other 4 studies including 2 used human NSPC and 2 used mice NSPC, and 3 of them used immunosuppression. Of all studies, there were 14 studies combined with other treatment methods. In almost all studies, NSPCs were stereotactically transplanted, only 1 was intranasally transplanted and 3 studies were intra-cerebroventricular injection. Information on study characteristics, study quality and publication bias is shown in Tables 1, 2, Additional files 3: Table S2, and 2: Fig. S1.

**Table 1:** Characteristics of mice trials

References	Location	Animal sex	Animal species	Animal year	Group	Type and source of NSPC	Way of administrated	Delivered location	Dose of NSPC	Immunosuppression	Assessment time	Parameter
Huang [15]	China	Female	APPswe/PS1dE9 mice	9 months	AD + PBS AD + NSC AD + nanoformulation-NSC AD + NEP–NSC	Mice NSC from hippocampus	Stereotactically transplanted	Hippocampus	1 × 10^5^	N	1 month 6 months	①②
Wu [16]	China	NA	Tg2576 mice	16 months	Tg + Vehicle Tg + NSC Tg + BDNF-NSC	Mice NSC from postnatal day 1 hippocampus	Stereotactically transplanted	Hippocampus	1 × 10^5^	N	8 weeks	①②④⑥
Lee [17]	Korea	male	ICR mice	NA	IBO acid + NSC (*n* = 9) IBO acid + PBS (*n* = 7) IBO acid + NGF-NSC (*n* = 9)	Human NSC from 14-week fetal brain	Stereotactically transplanted	Cortex	2 × 10^5^	N	4 weeks	①
McGinley [18]	USA	Male	APPswe/PS1dE9 mice	12 weeks	AD + vehicle (*n* = 10) AD + NSC (*n* = 10)	Human cortex-derived NSC	Stereotactically transplanted	Fimbria fornix	1.8 × 10^5^	Y	16 weeks	①②④⑤
Zhao [19]	China	male	SAMP8 mice	8 months	SAMP8 + sham operation (*n* = 10) SAMP8 + NSC (*n* = 10)	Mice NSC from embryo	Stereotactically transplanted	Hippocampus	5 × 10^5^	N	15 days	①⑥
Zhang [20]	China	male	APPswe/PS1dE9 mice	12 months	Tg + PBS (*n* = 10) Tg + NSC (*n *= 10)	Mice NSC	Stereotactically transplanted	Hippocampus	5 × 10^5^ to 1 × 10^6^	N	10 weeks	①④
Zhang [21]	China	NA	APPswe/PS1dE9mice	12 months	Tg + vehicle (*n* = 20) Tg + NSC (*n* = 20)	Mice NSC from 14 days embryo	Stereotactically transplanted	Hippocampus	2.5–5 × 10^6^	N	5 weeks 10 weeks	①
Mathew [22]	USA	NA	APP/PS1/tau 3 × Tg AD mice	18 months	3xTg-AD + vehicle (*n* = 9) 3xTg-AD + NSC (*n* = 18)	Mice NSC (postnatal day 1)	Stereotactically transplanted	Hippocampus	1 × 10^5^	N	1 month	①
Chen [23]	China	Male	APP/PS1/tau 3 × Tg AD mice	12 months	3xTg-AD + PBS (*n* = 10) 3xTg-AD + NSC (*n* = 10)	Mice NSC from hippocampus and subependymal zone of 12.5 days fetal brain	Stereotactically transplanted	Hippocampus	2 × 10^6^	N	8 weeks	①
Lu [24]	China	male	APPswe/PS1dE9 mice	3.5 months	AD + saline AD + NSC	Human NSC from hippocampus of 6–8-week embryos	Intranasally transplanted	Nasal cavity	1 × 10^6^, 4 times	Y	3 months 4 months	①②④⑤⑥
Zhang [25]	China	NA	Tg-tau mice	40 weeks	Tg + PBS (*n* = 11) Tg + NSC (*n* = 11)	Mice NSC from hippocampus on postnatal day1	Stereotactically transplanted	Hippocampus	2 × 10^5^	N	4 weeks	①③
Zhang [26]	China	male	APPswe/PS1dE9 mice	12 months	AD + PBS (*n* = 20) AD + NSC (*n* = 20)	Mice NSC from embryonic day 14	Stereotactically transplanted	Hippocampus	5 × 10^5^to 1 × 10^6^	N	10 weeks	①②⑤
Zhang [27]	China	NA	APPswe/PS1dE9 mice	10 months	AD + Vehicle (*n* = 15) AD + NSC (*n* = 15)	Mice NSC from embryonic day 14	Stereotactically transplanted	Hippocampus	1 × 10^6^	N	8 weeks	①④
Zhang [28]	China	NA	APPswe/PS1dE9 mice	12 months	AD + Vehicle (*n* = 20) AD + NSC (*n* = 20)	Mice NSC from embryonic day 14	Stereotactically transplanted	Hippocampus	5 × 10^5^ to 1 × 10^6^	N	8 weeks	①②④⑥
Zhou [29]	China	male	SAMP8 mice	8 months	SAMP8 control (*n* = 10) SAMP8 + NSC (*n* = 10) SAMP8 SAMP8 + NSC + HuangDiSan (*n* = 10)	Mice NSC from embryonic day 12–16	Stereotactically transplanted	Hippocampus	1 × 10^6^	N	15 days	①④
Ofra [30]	Israel	male	APPswe/PS1dE9 mice	11 months	AD + sham AD + NPC AD + IL-1ra-NPC	Mice NPC	Stereotactically transplanted	Hippocampus	4000 spheres	N	1 month	①②⑥
Armijo [31]	USA	male and female	APP/PS1/tau 3 × Tg AD mice	17 months	3xTg-AD + PBS 3xTg-AD + NPC	Mice NPC from tail-tip fibroblasts	Stereotactically transplanted	Hippocampus	5 × 10^5^	N	1 month 2 months	②③
Lee [32]	Korea	NA	NSE/APPsw transgenic mice	13 months	APP + Vehicle APP + NSC	Human NSC from 13-week fetal brain	ICV	Lateral ventricles	5 × 10^5^	Y	6 weeks 12 weeks	①②③④⑤⑥
Li [33]	China	male and female	APPswe/PS1dE9 mice	12 months	AD + Vehicle (*n* = 10) AD + NSC (*n* = 10)	Mice NSC from embryonic day 14	Stereotactically transplanted	Hippocampus	2.5–5 × 10^6^	N	3 weeks	①⑤⑥
Li [34]	China	NA	APPswe/PS1dE9 mice	NA	AD + Saline AD + NSC AD + CSeM/let-7b NPs-NSC	Mice NSC from hippocampus	Stereotactically transplanted	NA	NA	N	30 days	①⑥
Lilja [35]	Sweden	male and female	Tg2576 Mice	6–9 months	Tg + Vehicle + saline (*n* = 9) Tg + NSC + saline (*n* = 9) Tg + NSC + JN403 (*n* = 5) Tg + NSC + ( +)-phenserine (*n* = 7)	Human NSC	Stereotactically transplanted	Hippocampus	2.5 × 10^4^	N	5 weeks	①②
Park [36]	Korea	NA	APPswe/PS1dE9 mice	18 months	AD + saline (*n* = 10) AD + NSC (*n* = 10) AD + CHAT-NSC (*n* = 10)	Human NSC from 15-week fetal brain	ICV	Lateral ventricles	1 × 10^6^	N	4 weeks	①②⑥

*NA* not reported, *AD* Alzheimer’s disease, *NSC* neural stem cell, *NPC* neural progenitor cell, *N* No, *Y* Yes, *IBO acid* ibotenic acid, *ICV *intra-cerebroventricular injection, ① Morris water maze test, ② A*β* deposition, ③ p-tau level, ④ synaptic density, ⑤ anti-inflammatory effect, ⑥ brain-derived neurotrophic factor level

**Table 2:** Characteristics of rat trials

References	Location	Animal sex	Animal species	Animal weight	Animal year	Group	Type and source of NSPC	Way of administrated		Dose of NSPC	Immunosuppression	Assessment time	Parameter
Park [37]	Korea	Male	SD rat	220–230 g	NA	AF64A (*n* = 15) AF64A + NSC (*n* = 15) AF64A + ChAT NSC (*n* = 15)	Human NSC from 15-week fetal brain	ICV	Right ventricle	1 × 10^6^	N	4–5 weeks 8–9 weeks	MWM
Moghadam [38]	Iran	Male	SD rat	about 300 g	NA	nbM lesion + vehicle (*n* = 6) nbM lesion + NPC (*n* = 6)	Mice NPC differentiation from ESC	Stereotactically transplanted	Right nbM	2 × 10^5^	Y	4 weeks	MWM
Tang [39]	China	Male	Wistar rat	200–250 g	3–4 months	A*β* (*n* = 10) A*β* + NPC (*n* = 10)	Mice NPC from embryonic fibroblasts	Stereotactically transplanted	Hippocampus	NA	Y	4 weeks 16 weeks	MWM
Wu [40]	Japan	Male	Wistar rat	270–290 g	NA	OA (n = 12) OA + NSC (*n* = 12) OA + NSC-hNGF-eGFP (*n* = 12)	Rat NSC from 17-day rat forebrain cerebral cortex	Stereotactically transplanted	Hippocampus and cerebral cortex	2 × 10^5^	N	30 days	MWM
Chen [41]	China	Male	SD rat	200–250 g	NA	IgG-saporin (*n* = 8) IgG-saporin + NSC (*n* = 8) IgG-saporin + NSC + NGF-PE-PLGA-NPs (*N* = 8)	Rat NSC from embryonic day 13.5–15.5	Stereotactically transplanted	Hippocampus and basal forebrain	NA	N	4 weeks	MWM
Cui [42]	China	Female	SD rats	NA	NA	A*β* (*n* = 15) A*β* + NSC (*n* = 15) A*β* + NSC + DSP (*n* = 15)	Rat NSC from hippocampus on postnatal day1	Stereotactically transplanted	Hippocampus	5 × 10^5^	N	4 weeks	MWM
Hu [43]	China	Female	SD rats	NA	8 weeks	A*β* (*n* = 18) A*β* + NSC (*n* = 18) A*β* + NSC + ASI (*n* = 18)	Rat NSC from embryonic day 14	Stereotactically transplanted	Hippocampus	1 × 10^5^	N	4 weeks	MWM
Shaymaa [44]	Egypt	Male	SD rats	200–250 g	3 months	IBO acid (*n* = 10) IBO acid + NSC (*n* = 10) IBO acid + NSC + ROO (*n* = 10)	Human adult OBNSCs	Stereotactically transplanted	Hippocampus	2.4 × 10^5^	Y	7 weeks	MWM

*NA* not reported, *SD rat* Sprague Dawley rat, *OA* okadaic acid, *IBO acid* Ibotenic acid, *NSC* neural stem cell, *NPC* neural progenitor cell, *N* no, *Y* yes, *OBNSCs* olfactory bulb neuronal stem cells, *DSP* designer self-assemble peptide, *ROO* rosemary oil, *MWM* Morris water maze, *ICV* intra-cerebroventricular injection, *nbM* nucleus basalis of Meynert

### Cognitive function

**Fig. 3:**
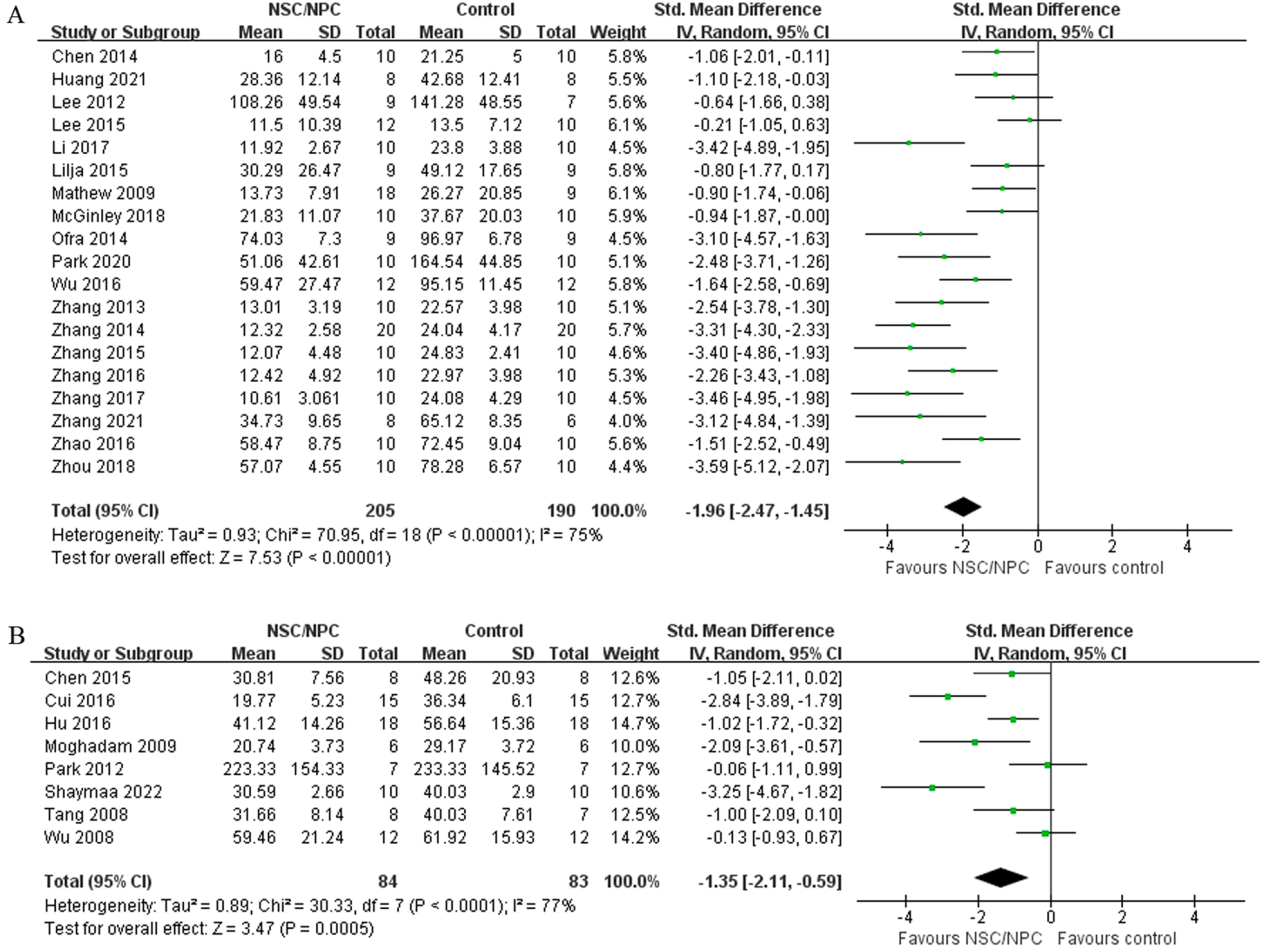
Forest plot shows the mean effect size and 95% confidence interval (CI) for cognitive function of mice studies **A** and rat studies **B** between NSPC treatment group and control group

Cognitive function was assessed by Morris water maze (MWM), and we extracted the data of escape latency from the last day of the learning phase. Nineteen of mice studies [15–23, 25–30, 32, 33, 35, 36] included MWM testing, we used a random-effect model to compare NSPC group (205 mice) and control group (190 mice), and the analysis showed that compared with the control group, NSPC could improve cognitive function apparently (SMD =  − 1.96, 95% CI − 2.47 to  − 1.45, *I*^2^ = 75%, *P* < 0.00001) (Fig. 3a). Eight rat studies [37–44] included MWM texting, we also used a random-effect model, and the outcome showed that cognitive function compared with control group (83 rats) and NSPC group (84 rats) improved apparently (SMD =  − 1.35, 95% CI − 2.11 to  − 0.59, *I*^2^ = 77%, *P* = 0.0005)(Fig. 3b).

### Pathological features

#### Aβ deposition

**Fig. 4:**
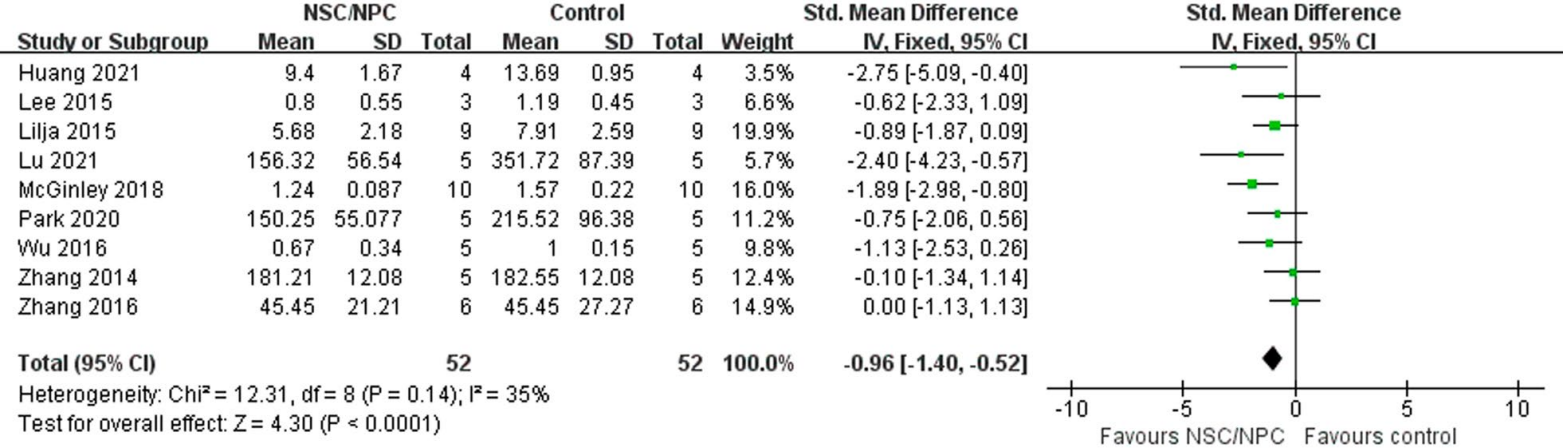
Forest plot shows the mean effect size and 95% confidence interval (CI) for A*β* deposition of mice studies between NSPC treatment group and control group

In mice studies, 9 studies [15, 16, 18, 24, 26, 28, 32, 35, 36] reported the difference of NSPC group (52 mice) and control group (52 mice) about A*β* deposition. We used a fixed-effect model for low heterogeneity (*P* = 0.14, *I*^2^ = 35%). Meta-analyses showed that A*β* deposition after NSPC treatment was significantly lower than AD models (SMD =  − 0.96, 95% CI − 1.40 to  − 0.52, *P* < 0.0001) (Fig. 4).

#### Synaptic density

**Fig. 5:**
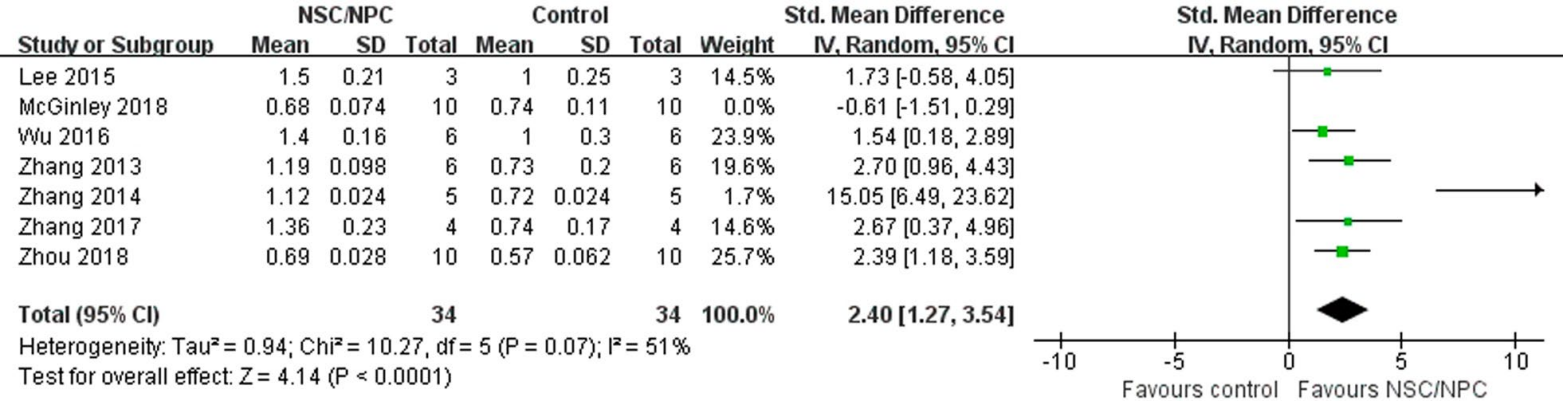
Forest plot shows the mean effect size and 95% confidence interval (CI) for synaptic density of mice studies between NSPC treatment group and control group

We used synaptophysin (SYP) expression to evaluate synaptic density to ensure if synaptic loss had been ameliorated, and 7 mice studies [16, 18, 20, 27–29, 32] reported it. Because of high heterogeneity (*P* < 0.00001, *I*^2^ = 82%) (Additional files 4: Fig. S2), we used a random-effect model, which showed that SYP expression of NSPC group (44 mice) was significantly higher than control group (44 mice), suggesting that NSPC promotes synaptic density recovery (SMD = 2.02, 95% CI 0.50–3.55, *P* = 0.009). Sensitivity analysis showed that high heterogeneity could be explained by the work of McGinley et al. [18]. After it was excluded, the level of heterogeneity decreased (*P* = 0.07, *I*^2^ = 51%) (Fig. 5).

#### Anti-inflammatory effect

**Fig. 6:**
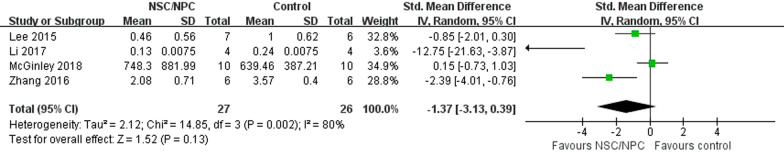
Forest plot shows the mean effect size and 95% confidence interval (CI) for anti-inflammatory effect of mice studies between NSPC treatment group and control group

IL-1*β* expression was used to assess anti-inflammatory effect of NSPC treatment, and 4 mice studies [18, 26, 32, 33] reported it. A random-effects model was used for the analysis because of the high heterogeneity (*P* = 0.002, *I*^2^ = 80%), and the results indicated that IL-1*β* expression did not change significantly (SMD =  − 1.37, 95% CI − 3.13 to 0.39, *P* = 0.13) (Fig. 6).

#### P-tau

**Fig. 7:**
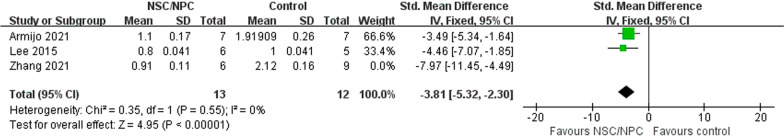
Forest plot shows the mean effect size and 95% confidence interval (CI) for p-tau level of mice studies between NSPC treatment group and control group

A total of 3 mice studies [25, 31, 32] compared p-tau level between NSPC group (19 mice) and control group (21 mice), and we used a random-effects model for the analysis because of the high heterogeneity (*P* = 0.08, *I*^2^ = 60%). The outcome showed that the *p*-tau level of NSPC group is lower (SMD =  − 4.94, 95% CI  − 7.29 to − 2.59, *P* < 0.0001) (Additional files 5: Fig. S3). Sensitivity analysis showed that high heterogeneity could be explained by the work of Zhang et al. [25]. After it was excluded, the level of heterogeneity decreased (*P* = 0.55, *I*^2^ = 0%) (Fig. 7). But due to the small number of data, we need more studies to make a conclusion.

### Brain-derived neurotrophic factor (BDNF)

**Fig. 8:**
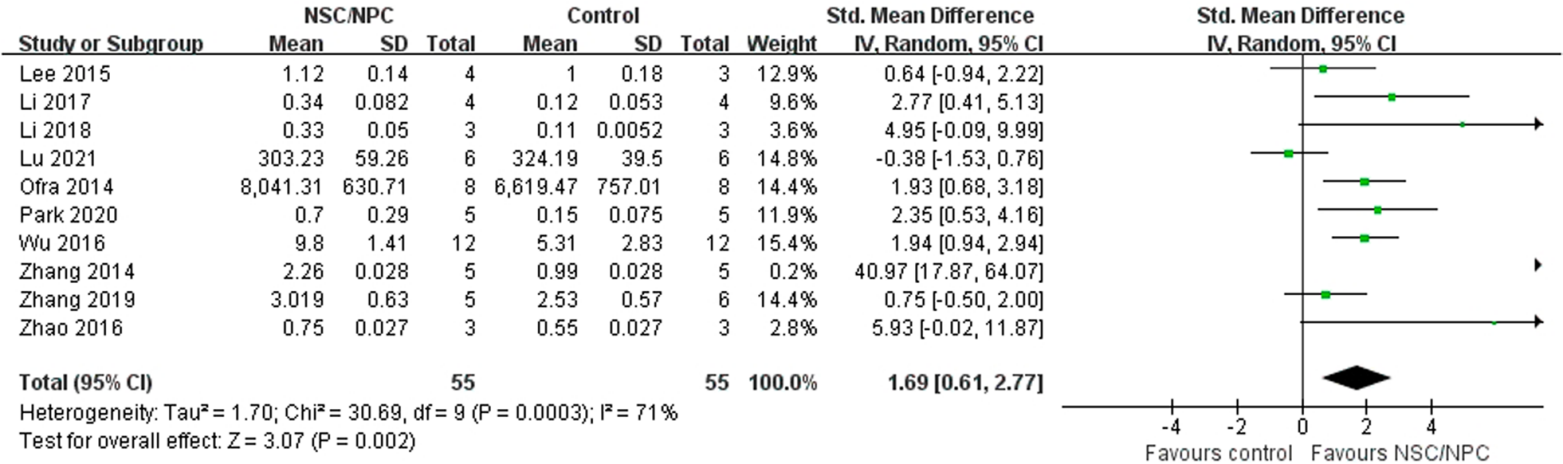
Forest plot shows the mean effect size and 95% confidence interval (CI) for BDNF level of mice studies between NSPC treatment group and control group

A total of 10 mice studies [16, 19, 24, 28, 30, 32–34, 36, 45] reported BDNF level, we used a random-effect model to compare BDNF level between NSPC group (55 mice) and control group (55 mice) because of high heterogeneity (*P* = 0.0003, *I*^2^ = 71%). BDNF level of NSPC group was higher than control group (SMD = 1.69, 95% CI 0.61–2.77, *P* = 0.002) (Fig. 8).

### Effect of NSPC combined with other treatment

**Fig. 9:**
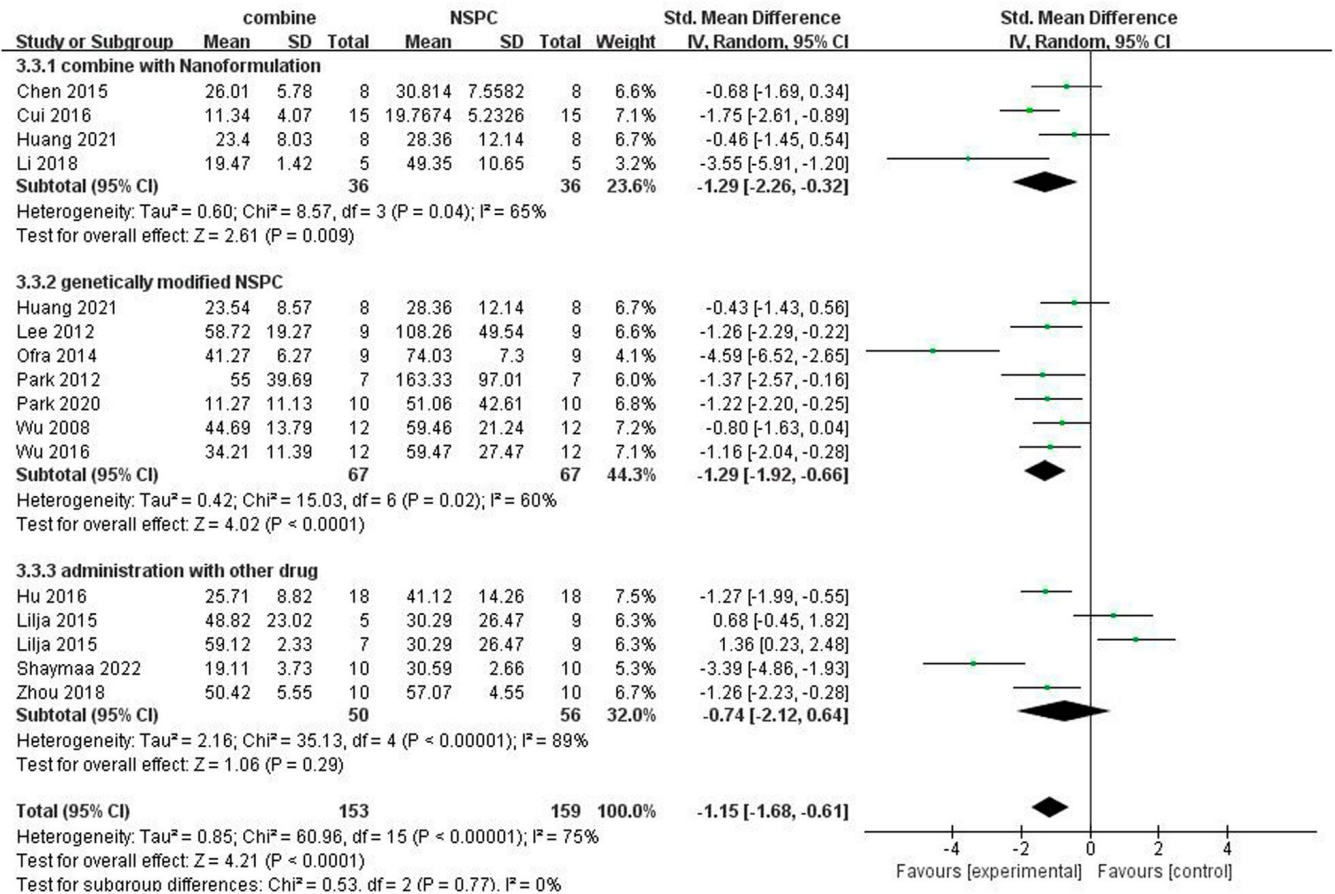
Forest plots of subgroup analysis by effect of NSPC combined with other treatment for cognitive function in preclinical rodent models

A total of 14 studies used NSPC combined with other treatment, we divided them into 3 group: a: NSPC combined with nanoformulation (4 studies) [15, 33, 41, 42], b: genetically modified NSPC (7 studies) [15–17, 30, 36, 37, 40], and c: NSPC administration with other drug (4 studies with 5 drugs) [29, 35, 43, 44]. We used a subgroup analysis to compare the effect between combination group with NSPC group on cognitive function (Fig. 9). The outcome proved that both combined with nanoformulation (SMD =  − 1.29, 95% CI  − 2.26 to − 0.32, *I*^2^ = 65%, *P* = 0.009) and genetically modified NSPC (SMD =  − 1.29, 95% CI − 1.92 to − 0.66, *I*^2^ = 60%, *P* < 0.0001) can enhance the effect of NSPC therapy. But consolidated analysis suggested that there was no statistically significant difference in cognitive function between NSPC treatment and NSPC administration with other drug (SMD =  − 0.74, 95% CI  − 2.12 to 0.64, *I*^2^ = 89%, *P* = 0.29).

### Effect of NSPC xenogeneic and allogeneic transplant for cognitive function

Nineteen of mice studies and 8 rat studies included MWM testing, and we divided them into 2 groups: xenogeneic transplant group (7 studies) [17, 18, 32, 35–37, 44] and allogeneic transplant group (20 studies) [15, 16, 19–23, 25–30, 33, 38–43]. We used a subgroup analysis to evaluate the effect of NSPC xenogeneic transplant and allogeneic transplant on cognitive function (Fig. 12). The outcome proved that both xenogeneic transplant (SMD =  − 1.10, 95% CI  − 1.86 to − 0.35, *I*^2^ = 73%, *P* = 0.004) and allogeneic transplant (SMD =  − 2.01, 95% CI  − 2.50 to  − 1.53, *I*^2^ = 74%, *P* < 0.00001) treatment could improve cognitive function apparently.

### Sensitivity analysis

To evaluate the stability of the results, we further performed a sensitivity analysis through the sequential omission of each study. For the pooled SMD, outcome of cognitive function, A*β* deposition and BDNF level were not significantly affected by any study.

## Discussion

Current treatments of AD are unable to achieve satisfactory therapeutic outcomes, so an effective and safe treatment is urgently required. We explored whether NSPC could be used to treat AD. Our meta-analysis of 30 studies made a comprehensive summary about the effect of NSPC therapy on the mice and rat model of AD. Pooled analyses confirmed that NSPC therapy could improve cognitive function in the preclinical models of AD. Our analysis also suggests that inject NSPC with nanoformulation and genetically modified boost the efficacy of NSPC treatment. Therefore, the present meta-analysis provides significant clues for human clinical trials on NSPC therapy.

Alzheimer’s disease is a progressive neurodegenerative disorder, which is a major cause of dementia [46], so we chose cognitive function as outcome indicate. The pathological features of AD include the presence of extracellular A*β*-containing senile plaques and intracellular hyperphosphorylated tau-containing NFT, neuroinflammation and synaptic loss, so we used A*β* deposition, synaptic density, anti-inflammatory effect and p-tau level as pathological indication. We found that BDNF was observed in several studies, so we analyzed the change of BDNF to evaluate the function of NSPC therapy.

**Fig. 10:**
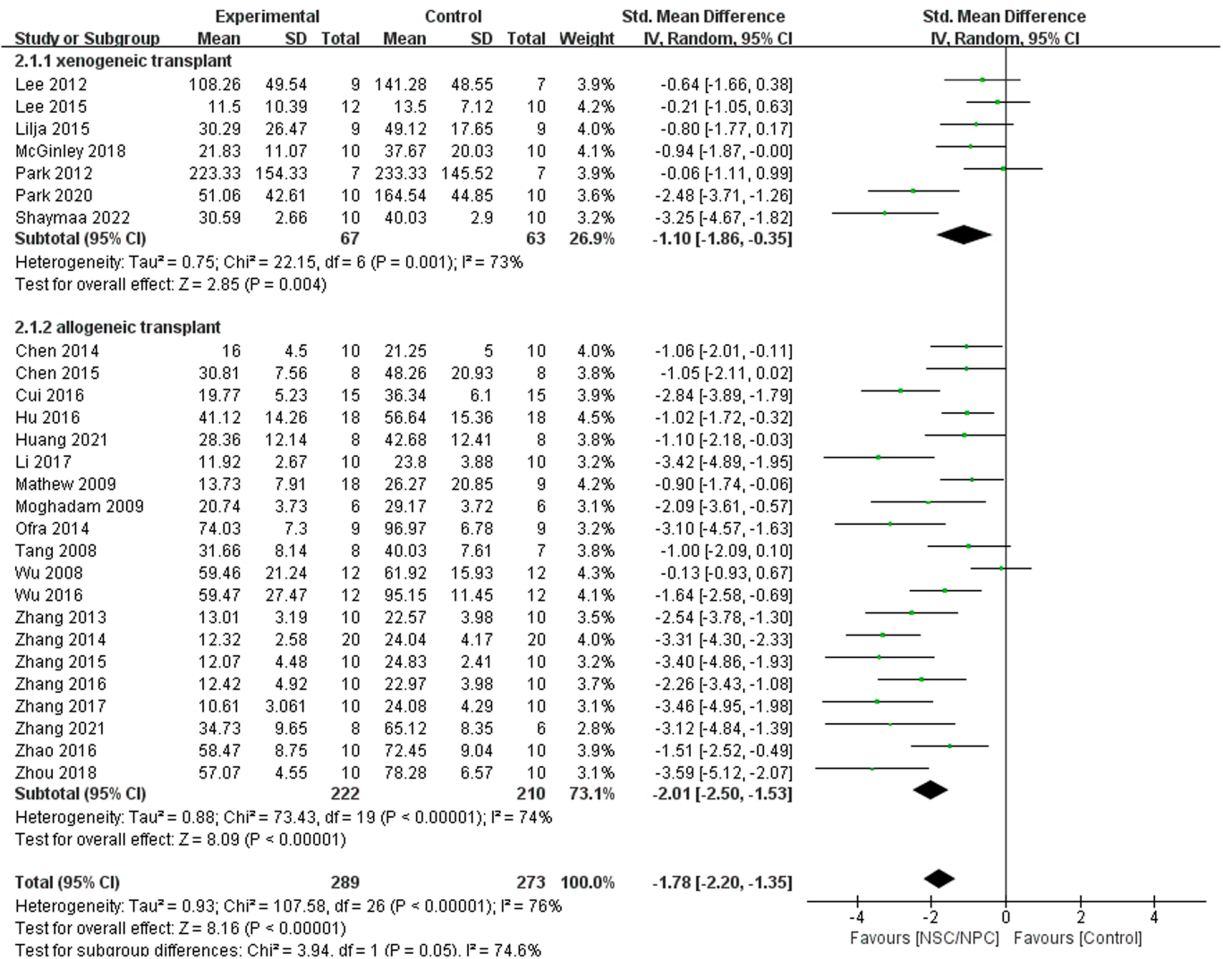
Forest plots of subgroup analysis by effect of NSPC allogeneic transplant and NSPC xenogeneic transplant for cognitive function in preclinical rodent models

**Fig. 11:**
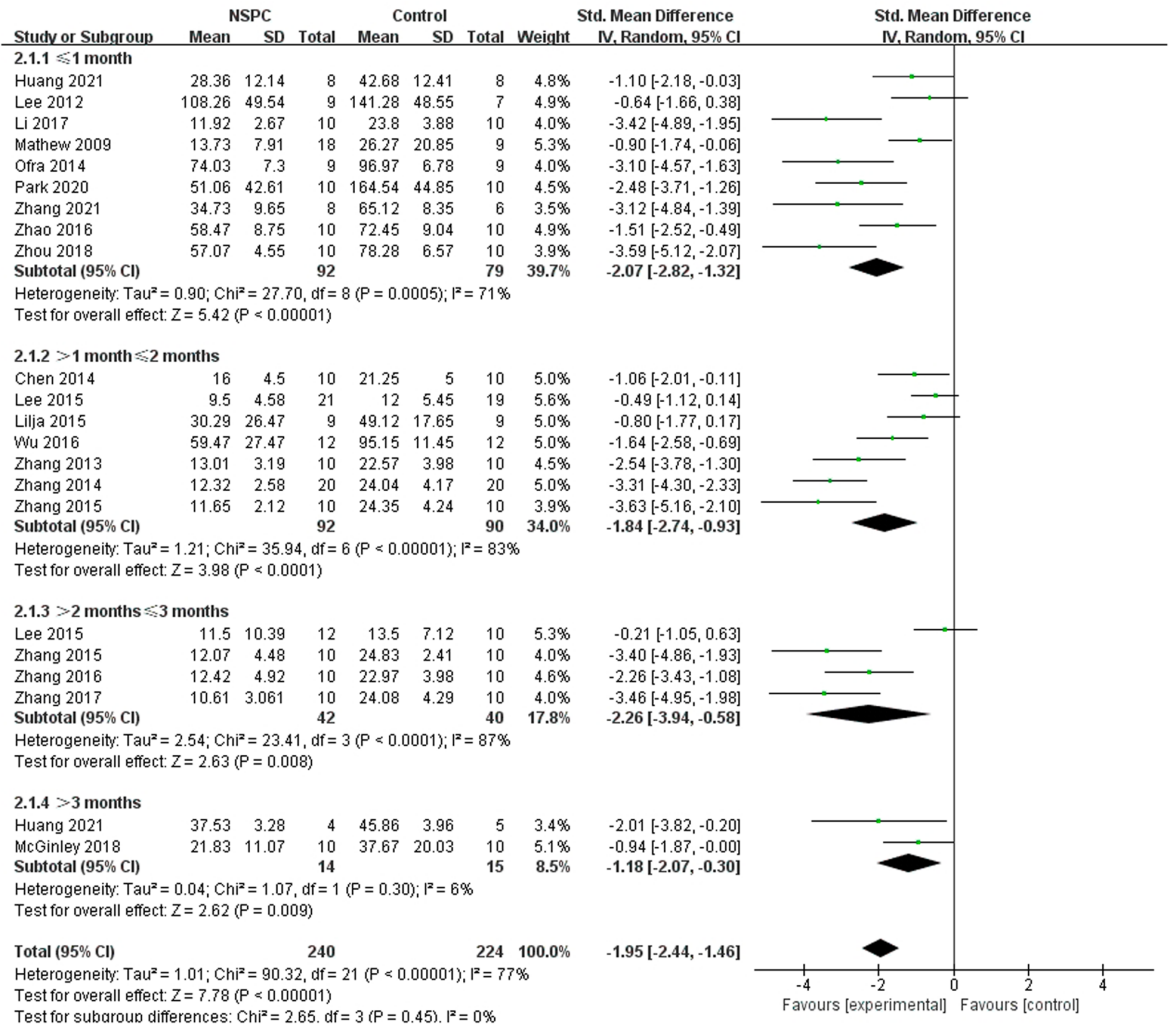
Forest plots of subgroup analysis by assessment time for cognitive function in mice model

Morris water maze (MWM) experiment is widely used in scientific research to assess the learning and memory of animals [47]. Almost all studies use MWM experiment as behavioral experiments to observe whether cognitive function has improved. In this analysis, we used the data of escape latency from the last day of the learning phase to evaluate the cognitive function. Compared to control group, almost all data of NSPC treatment group were lower, which means that NSPC therapy could improve the learning and memory function of AD model and ameliorate the deterioration of cognitive function. The subgroup analysis of assessment time in mice trials showed that after 3 months, NSPC therapy still has effectiveness (SMD =  − 1.18, 95% CI  − 2.07 to 0.30, *I*^2^ = 6%, *P* = 0.009) (Fig. 10). But in rat studies, after 1 month, NSPC does not work (SMD =  − 1.62, 95% CI − 4.74 to 1.50, *I*^2^ = 92%, *P* = 0.31) (Fig. 11).

**Fig. 12:**
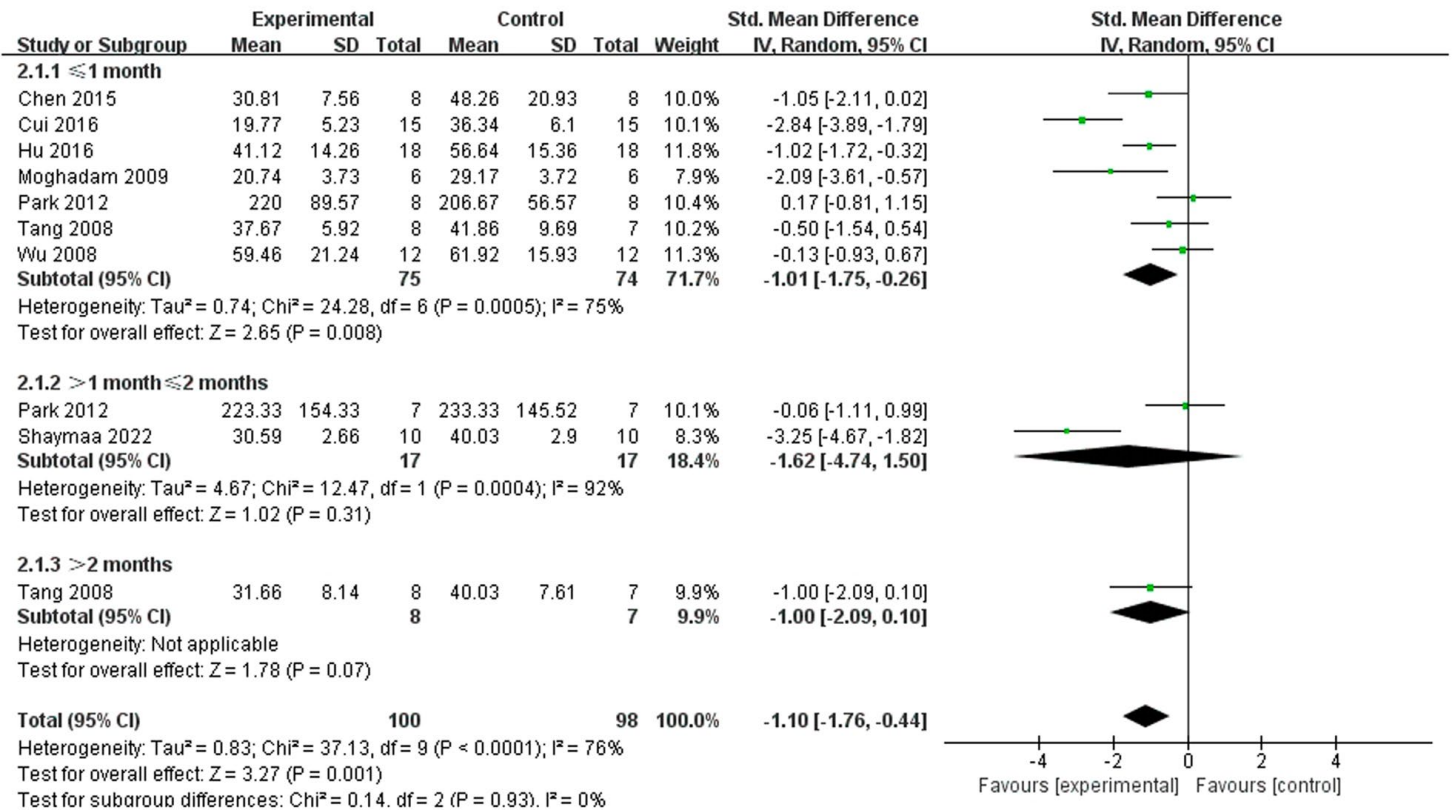
Forest plots of subgroup analysis by assessment time for cognitive function in rat model

A*β* is one of the key initiating factors of AD pathogenesis. Accumulation of A*β* results in loss of synapses, neuroinflammation and ultimately cognitive deficits [48]. Our analysis collected the data about A*β* expression of NSPC group and control group; compared to control group, A*β* deposition of NSPC group was significantly lower, so we can conclude that NSPC decreases A*β* accumulation. Tau proteins are microtubular neuronal proteins. The tau proteins have a microtubule binding domain, which is involved in polymerization and stabilization of the microtubule assembly to maintain the integrity of the cytoskeleton. Hyperphosphorylation results in decreased affinity of the tau proteins to microtubules. The hyperphosphorylated tau forms NFTs and gets deposited in the cytosol and can no longer perform the function of maintaining the structure of the cell [46]. Moreover, it would impair cognitive function. Of all studies, 3 studies [25, 31, 32] reported p-tau level and suggested that NSPC treatment would reduce p-tau aggregation (Fig. 12).

A synaptic damage in the neocortex and limbic system causes memory impairment and generally is observed at the early stages of AD [49]. SYP is a specific protein on the membrane of synaptic vesicles, which may be involved in the formation of synaptic vesicles and dendrite spine. Here, we used SYP to evaluate synaptic density. The data of NSPC group were higher than control group, and it can prove that NSPC transplantation enhances synaptic density, attenuated the synaptotoxic properties of A*β* and promoted synaptic plasticity [32]. Electrophysiological recording of 2 studies [31, 45] also proved that NSPC transplantation promoted synaptic plasticity.

Many studies now point to the involvement of neuroinflammation playing a fundamental role in the progression of the neuropathological changes that are observed in AD [50]. Unlike other risk factors and genetic causes of AD, neuroinflammation is not typically thought to be causal on its own but rather a result of one or more of the other AD pathologies or risk factors associated with AD and serves to increase the severity of the disease by exacerbating *β*-amyloid and tau pathologies [51, 52]. IL-1*β* has been described as a “master regulator” within the brain inflammatory cascade, and disruptions to IL-1*β* can delay the onset of neuroinflammation and neurodegeneration [53]. We used IL-1*β* expression to evaluate neuroinflammation, though the data we collected of NSPC group were lower than control group, and there was no statistical significance between two groups. One study quantified the density of microglia and astrocytes and proved that NSCs transplantation reduced the density of astrocytes and microglia, suggesting that NSCs inhibit neuroinflammation [24, 54].

In the brain, BDNF is expressed by glutamatergic neurons and glial cells, such as astrocytes isolated from the cortex and hippocampus [54, 55]. BDNF is a neurotrophin that modulates the survival of stem cells and progenitors, neurogenesis and neuronal differentiation, the branching and survival of differentiated neurons and the formation and maturation of the dendritic spine and synapses. Thus, BDNF influences learning and memory [56]. And our analysis demonstrated that NSPC treatment could improve BDNF level to ameliorate the condition of AD.

## Limitations

Several potential limitations of our meta-analysis should be considered. First, although we performed stratified and sensitivity analyses, the heterogeneity among studies could not be remarkably reduced. This may influence the stability of the results. Second, data of A*β* deposition, SYP expression, tau level and more indicators were lacked in several studies, and role of NSPC in AD alleviation requires further evaluation. Third, our meta-analysis only observed mice and rat models, which are not able to well simulate the physical conditions of human suffered from AD.

## Conclusion

The data of our meta-analysis revealed, NSPC transplantation may enhance the cognitive function and reduce AD burden, while the nanoformulation and genetically modification may promote the effect of NSPC therapy. Which would provide the theoretical foundation and guide for clinical trials of NSPC for AD. Both xenogeneic and allogeneic transplant of NSPC could improve the cognitive function of AD animals. More animal studies and human trials are needed for further investigation.

## Supplementary Information

**Additional file 1: Table S1.** The detailed search strategy.

**Additional file 2: Fig. S1.** Evaluation of publication bias. Funnel plots for Aβ deposition (**A**), mice cognitive function (**B**), rat cognitive function (**C**) and BDNF (**D**).

**Additional file 3: Table S2.** SYRCLE’s RoB tool for each experimental animal studies.

**Additional file 4: Fig. S2**. Forest plot for synaptic density of mice studies between NSPC treatment group and control group. It had high heterogeneity before the work of McGinley et al. was excluded.

**Additional file 5: Fig. S3.** Forest plot for p-tau level of mice studies between NSPC treatment group and control group. Because of high heterogeneity, we used a random-effect model.

## Data Availability

All supporting data are included in the article.

## Abbreviations

AD: Alzheimer’s disease
NSPC: Neural stem/progenitor cell
SMD: Standard mean difference
CI: Confidence interval
A*β*: Amyloid-*β*
NFT: Neurofibrillary tangles
FAD: Familial Alzheimer disease
SAD: Sporadic Alzheimer disease
AChEIs: Acetylcholinesterase inhibitors
NMDAR: *N*-Methyl-d-aspartate receptor
DBS: Deep brain stimulation
rTMS: Repetitive transcranial magnetic stimulation
MSC: Mesenchymal stem cell
iPSC: Induced pluripotent stem cell
NSC: Neural stem cell
ESC: Embryonic stem cell
MeSH: Medical subject headings
SD rat: Sprague Dawley rat
OA: Okadaic acid
IBO acid: Ibotenic acid
OBNSCs: Olfactory bulb neuronal stem cells
DSP: Designer self-assemble peptide
ROO: Rosemary oil
MWM: Morris water maze
SYP: Synaptophysin
BDNF: Brain-derived neurotrophic factor
ICV: Intra-cerebroventricular injection
nbM: Nucleus basalis of Meynert
