# Supplementary material for: Neural stem/progenitor cell therapy for Alzheimer disease in preclinical rodent models: a systematic review and meta-analysis
Source: Stem Cell Res Ther. 2023 Jan 5;14:3. doi: 10.1186/s13287-022-03231-1 (PMC9814315; doi:10.1186/s13287-022-03231-1)
Supplement: Supplementary file 1 — Additional file 1: Table S1. The detailed search strategy. [file 13287_2022_3231_MOESM1_ESM.docx]

| Electronic databases | Search | Search strategy |  | Results |
| --- | --- | --- | --- | --- |
| **PUBMED** | #1 | "Alzheimer Disease"[Mesh] |  | 113,748 |
|  | #2 | (((("Alzheimer Disease"[Mesh]) OR (Alzheimer Dementia)) OR (Dementia, Alzheimer)) OR (Alzheimer's Disease)) OR (Alzheimer Syndrome) |  | 196,775 |
|  | #3 | "Neural Stem Cells"[Mesh] |  | 13,478 |
|  | #4 | ((("Neural Stem Cells"[Mesh]) OR (Neural Stem Cell)) OR (Neural Progenitor Cell)) OR (neural stem/progenitor cell) |  | 43,076 |
|  | #5 | ("Mice"[Mesh]) OR "Rats"[Mesh] |  | 3,276,499 |
|  | #6 | ((("Mice"[Mesh]) OR "Rats"[Mesh]) OR (mouse)) OR (rat) |  | 3,609,055 |
|  | #7 | #2 AND #4 AND #6 |  | **711** |
| **EMBASE** | #1 | ('alzheimer disease'/exp OR 'alzeimer`s disease' OR 'alzheimer dementia' OR 'alzheimer syndrome' OR 'dementia, alzheimer') AND ('neural stem cell'/exp OR 'neural progenitor cell' OR 'neural stem cells' OR 'neural stem/progenitor cell') |  | 1,855 |
|  | #2 | mice OR mouse OR rat OR rats |  | [4,322,861](https://www.embase.com/) |
|  | #3 | #1 AND #2 |  | **688** |
| **Cochrane Library** | #1 | MeSH descriptor: [Alzheimer Disease] explode all trees |  | 3,866 |
|  | #2 | (Alzheimer Dementia):ti,ab,kw |  | 5,437 |
|  | #3 | (Dementia, Alzheimer):ti,ab,kw |  | 5,437 |
|  | #4 | (Alzheimer's Disease):ti,ab,kw |  | 12,192 |
|  | #5 | (Alzheimer Syndrome):ti,ab,kw |  | 472 |
|  | #6 | #1 or #2 or #3 or #4 or #5 |  | 12,600 |
|  | #7 | MeSH descriptor: [Neural Stem Cells] explode all trees |  | 6 |
|  | #8 | (Neural Stem Cell):ti,ab,kw |  | 135 |
|  | #9 | (Neural Progenitor Cell):ti,ab,kw |  | 25 |
|  | #10 | #7 or #8 or #9 |  | 142 |
|  | #11 | MeSH descriptor: [Mice] explode all trees |  | 1,281 |
|  | #12 | MeSH descriptor: [Rats] explode all trees |  | 1,124 |
|  | #13 | (mouse):ti,ab,kw |  | 5,492 |
|  | #14 | (rat):ti,ab,kw |  | 2,453 |
|  | #15 | #11 or #12 or #13 or #14 |  | 8,271 |
|  | #16 | #6 and #10 and #15 |  | **4** |
| **Web of Science** | #1 | ((((TS=(Alzheimer Disease)) OR TS=(Alzheimer Dementia)) OR TS=(Alzheimer Syndrome)) OR TS=(Alzheimer's Disease)) OR TS=(Dementia, Alzheimer) |  | 188,036 |
|  | #2 | (((TS=(Neural Stem Cells)) OR TS=(Neural Stem Cell)) OR TS=(Neural Progenitor Cell)) OR TS=(Neural Progenitor Cell) |  | 51,092 |
|  | #3 | (((TS=(Mice)) OR TS=(mouse)) OR TS=(Rats)) OR TS=(rat) |  | 3,361,068 |
|  | #4 | #1 AND #2 AND #3 and Alzheimer Disease or Mice or Neural Stem Cells or Rats (MeSH 主题词) and 综述论文 (排除 – 文献类型) |  | **695** |

**Table S1. The detailed search strategy**
