# Supplementary figures and images for: Neural stem/progenitor cell therapy for Alzheimer disease in preclinical rodent models: a systematic review and meta-analysis
Source: Stem Cell Res Ther. 2023 Jan 5;14:3. doi: 10.1186/s13287-022-03231-1 (PMC9814315; doi:10.1186/s13287-022-03231-1)

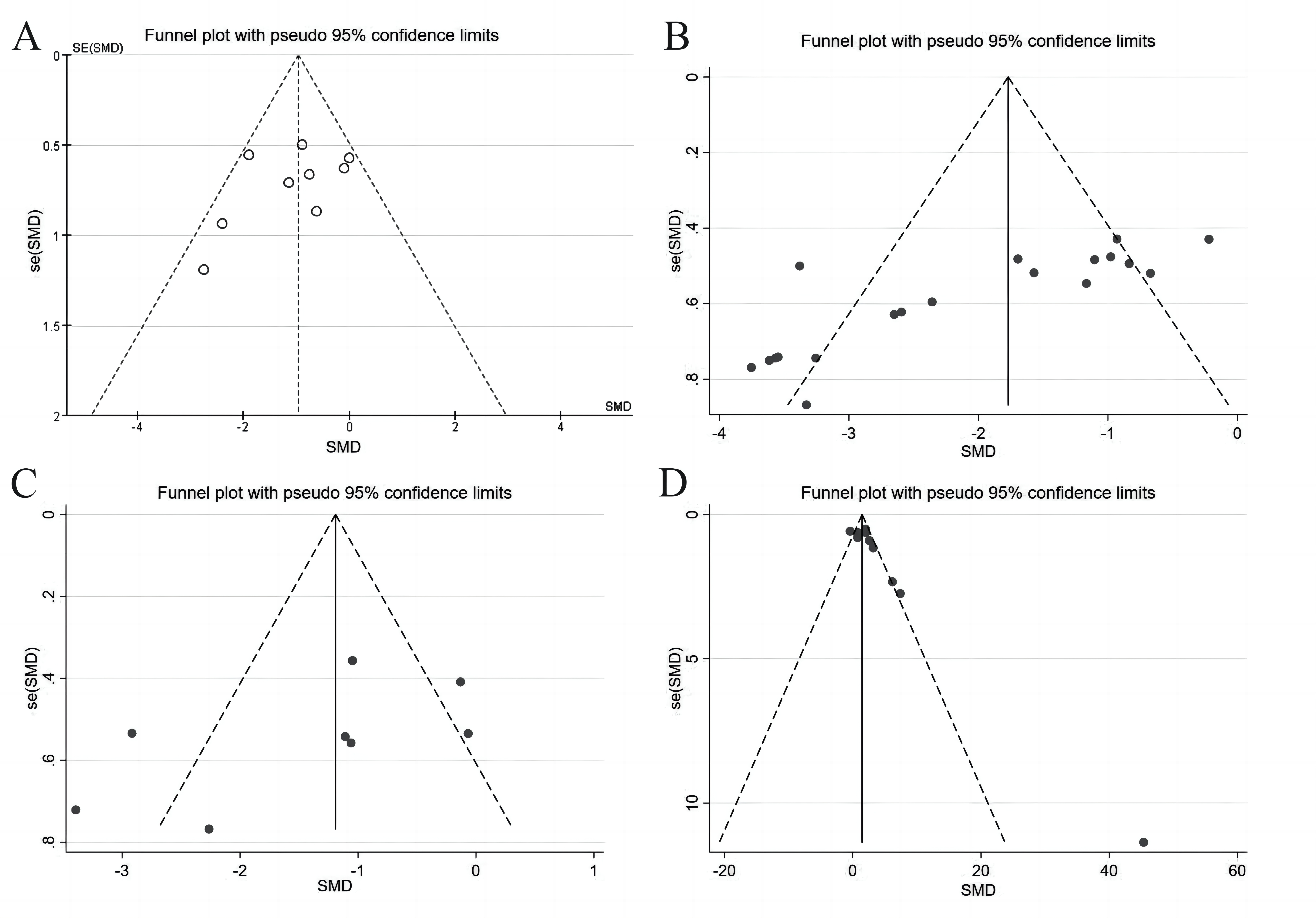

Supplement: Supplementary file 2 — Additional file 2: Fig. S1. Evaluation of publication bias. Funnel plots for Aβ deposition (A), mice cognitive function (B), rat cognitive function (C) and BDNF (D). [file 13287_2022_3231_MOESM2_ESM.jpg]

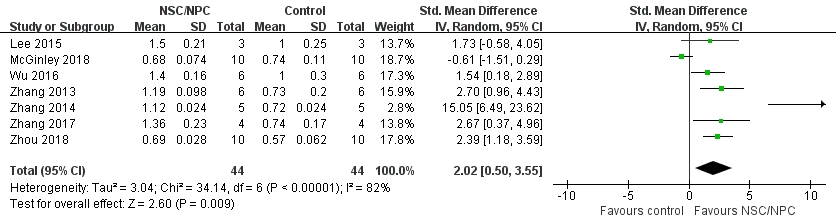

Supplement: Supplementary file 4 — Additional file 4: Fig. S2. Forest plot for synaptic density of mice studies between NSPC treatment group and control group. It had high heterogeneity before the work of McGinley et al. was excluded. [file 13287_2022_3231_MOESM4_ESM.jpg]

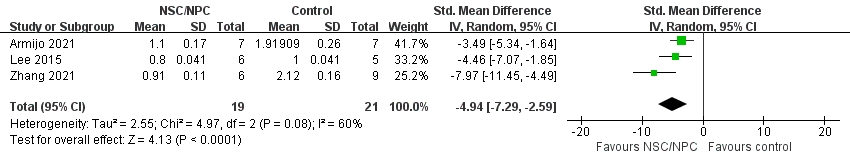

Supplement: Supplementary file 5 — Additional file 5: Fig. S3. Forest plot for p-tau level of mice studies between NSPC treatment group and control group. Because of high heterogeneity, we used a random-effect model. [file 13287_2022_3231_MOESM5_ESM.jpg]
