# Supplementary material for: Neural stem/progenitor cell therapy for Alzheimer disease in preclinical rodent models: a systematic review and meta-analysis
Source: Stem Cell Res Ther. 2023 Jan 5;14:3. doi: 10.1186/s13287-022-03231-1 (PMC9814315; doi:10.1186/s13287-022-03231-1)
Supplement: Supplementary file 3 — Additional file 3: Table S2. SYRCLE’s RoB tool for each experimental animal studies. [file 13287_2022_3231_MOESM3_ESM.docx]

**Table S2.** SYRCLE’s RoB tool for each experimental animal studies.

|  | SELECTION BIAS | | | PERFORMANCE BIAS | | DETECTION BIAS | | ATTRITION BIAS | REPORTING BIAS | OTHER  BIAS |
| --- | --- | --- | --- | --- | --- | --- | --- | --- | --- | --- |
|  | Random Sequence Generation | Baseline Characteristics | Allocation Concealment | Random Housing | Blinding (study Team) | Random Outcome Assessment | Blinding (Outcome Assessors) | Incomplete Outcome Data | Selective Outcome Reporting | Other  Source of  Bias |
| Huang  2021 | U | Y | U | U | U | U | U | Y | Y | U |
| Wu,2016 | U | Y | U | U | U | U | Y | Y | Y | U |
| Lee  2012 | U | Y | U | U | U | U | U | Y | Y | U |
| McGinley2018 | U | Y | U | U | U | U | U | Y | Y | U |
| Zhao  2016 | U | Y | U | U | U | U | U | Y | Y | U |
| Zhang  2017 | U | Y | U | U | U | U | U | Y | Y | U |
| Zhang  2015 | U | Y | U | U | U | U | U | Y | Y | U |
| Mathew  2009 | U | Y | U | U | U | U | Y | Y | Y | U |
| Chen  2014 | U | Y | U | U | U | U | U | Y | Y | U |
| Lu,  2021 | U | Y | U | U | U | U | U | Y | Y | U |
| Zhang  2021 | U | Y | U | U | U | U | U | Y | Y | U |
| Zhang  2016 | U | Y | U | U | U | U | U | Y | Y | U |
| Zhang  2013 | U | Y | U | U | U | U | U | Y | Y | U |
| Zhang  2014 | U | Y | U | U | U | U | U | Y | Y | U |
| Zhou  2018 | U | Y | U | U | U | U | U | Y | Y | U |
| Ofra  2014 | U | Y | U | U | U | U | U | Y | Y | U |
| Armijo  2021 | U | Y | U | U | U | U | Y | Y | Y | U |
| Lee,  2015 | U | Y | U | U | U | U | U | Y | Y | U |
| Li,  2017 | U | Y | U | U | U | U | U | Y | Y | U |
| Li,  2018 | U | Y | U | U | U | U | U | Y | Y | U |
| Lilja  2015 | U | Y | U | U | U | U | U | Y | Y | U |
| Park  2020 | U | Y | U | U | U | U | U | Y | Y | U |
| Park  2012 | U | Y | U | U | U | U | U | Y | Y | U |
| Moghadam2009 | U | Y | U | U | U | U | U | Y | Y | U |
| Tang,  2008 | U | Y | U | U | U | U | U | Y | Y | U |
| Wu,  2008 | U | Y | U | U | U | U | Y | Y | Y | U |
| Chen,  2015 | U | Y | U | U | U | U | Y | Y | Y | U |
| Cui,  2016 | U | Y | U | Y | U | U | Y | Y | Y | U |
| Hu,  2016 | U | Y | U | U | U | U | Y | Y | Y | U |
| Shaymaa,  2022 | U | Y | U | U | U | U | Y | Y | Y | U |

Y: low risk of bias N: high risk of bias U: unclear risk of bias
